# New insights into non-small cell lung cancer bone metastasis: mechanisms and therapies

**DOI:** 10.7150/ijbs.100960

**Published:** 2024-10-21

**Authors:** Man Xue, Li Ma, Pengpeng Zhang, Hui Yang, Zhaoxia Wang

**Affiliations:** 1 Department of Oncology, The Second Affiliated Hospital of Nanjing Medical University, Nanjing, China.; 2 Department of Lung Cancer, Tianjin Lung Cancer Center, National Clinical Research Center for Cancer, Key Laboratory of Cancer Prevention and Therapy, Tianjin's Clinical Research Center for Cancer, Tianjin Medical University Cancer Institute and Hospital, Tianjin, China.; 3 Department of Oncology, The Affiliated Jiangning Hospital of Nanjing Medical University, Nanjing, China.

**Keywords:** Non-small cell lung cancer, Bone metastasis, Signaling pathways, Bone-targeting agents

## Abstract

Bone metastasis is a common cause of death in patients with non-small cell lung cancer (NSCLC), with approximately 30-40% of NSCLC patients eventually developing bone metastases. Bone metastasis, especially the occurrence of skeletal-related events (SREs), significantly reduces overall survival (OS) and quality of life (QoL) in patients. Although bone-targeting agents (BTAs) have been shown to reduce SREs and improve QoL in NSCLC patients with bone metastases, the prognosis for these patients remains poor. Understanding the underlying molecular pathways of bone metastasis is crucial for the development of novel therapeutic approaches. Bone metastasis is a complex, multistep process that involves interactions between tumor cells and the bone microenvironment. The bone microenvironment provides a fertile soil for tumor cells, and crosstalk among various signaling pathways and secreted factors also plays a role in regulating the occurrence and progression of bone metastasis in NSCLC. In this article, we provide a comprehensive review of the process, regulatory mechanisms, and clinical treatment in NSCLC bone metastasis, with the hope of assisting with clinical treatment.

## Introduction

Lung cancer remains the leading cause of human cancer deaths worldwide^1^. According to 2024 tumor statistics, 125,707 cancer deaths are projected to occur in the United States, posing a serious threat to human survival and development^2^. Due to the insidious onset of lung cancer, more than 50% of patients are diagnosed at an advanced stage (stage IV), significantly affecting their prognosis. NSCLC accounts for more than 80% of all lung cancers and its deaths are mainly attributed to metastases. The skeleton is one of the most common sites of NSCLC metastases. Statistics indicate that 30-40% of NSCLC patients will eventually develop bone metastases, and 60% of these patients have bone metastases at the time of diagnosis^3^. In recent years, advancements in targeted therapy and immunotherapy have significantly prolonged the OS of metastatic NSCLC patients, potentially leading to a higher incidence of bone metastases. SEER studies have shown that lung cancer is the most common primary tumor in patients with bone metastases older than 25 years in 2015^4^.

Bone metastasis can lead to bone pain, pathological fractures, spinal cord compression, hypercalcemia, and other SREs that pose considerable challenges to patients' survival and QoL. Compared to NSCLC patients without bone metastases, patients with bone metastases have a worse prognosis, with a median OS of only 5 months and a 5-year survival rate of less than 5%^5^, ^6^. Bone metastasis also represents a substantial economic burden to patients and the healthcare system^7^. Currently, it is estimated that a total of more than 250,000 patients in the United States suffers from metastatic bone disease, with an estimated annual cost of $12 billion^8^. There is an urgent need for treatments targeting bone metastasis to reduce SREs, maximize improvements in patients' QoL, and increase survival rates.

Although bone metastasis is rarely curable, long-term control can be achieved with systemic therapy. Surgery and radiotherapy are local treatment modalities for bone metastasis, which can significantly alleviate bone pain and improve local function in patients. BTAs, such as bisphosphonates (BPs) and denosumab, can inhibit osteolytic lesions by inhibiting osteoclast-mediated bone resorption, and serve as fundamental treatment for osteolytic metastasis. Multiple studies have shown that BTAs not only demonstrate advantages in improving OS and delaying the occurrence of SREs, but also alleviate the economic burden^9^-^11^. Additionally, systemic treatments based on chemotherapy, targeted therapy, and immunotherapy also play crucial roles in the treatment of bone metastasis.

This article mainly explores the molecular mechanisms of bone metastasis in NSCLC, in order to block its occurrence from multiple targets. Furthermore, we review the main treatment modalities for NSCLC bone metastasis with the aim of providing assistance in the treatment of bone metastasis.

## Mechanisms of Non-Small Cell Lung Cancer Bone Metastasis

**Figure 1:**
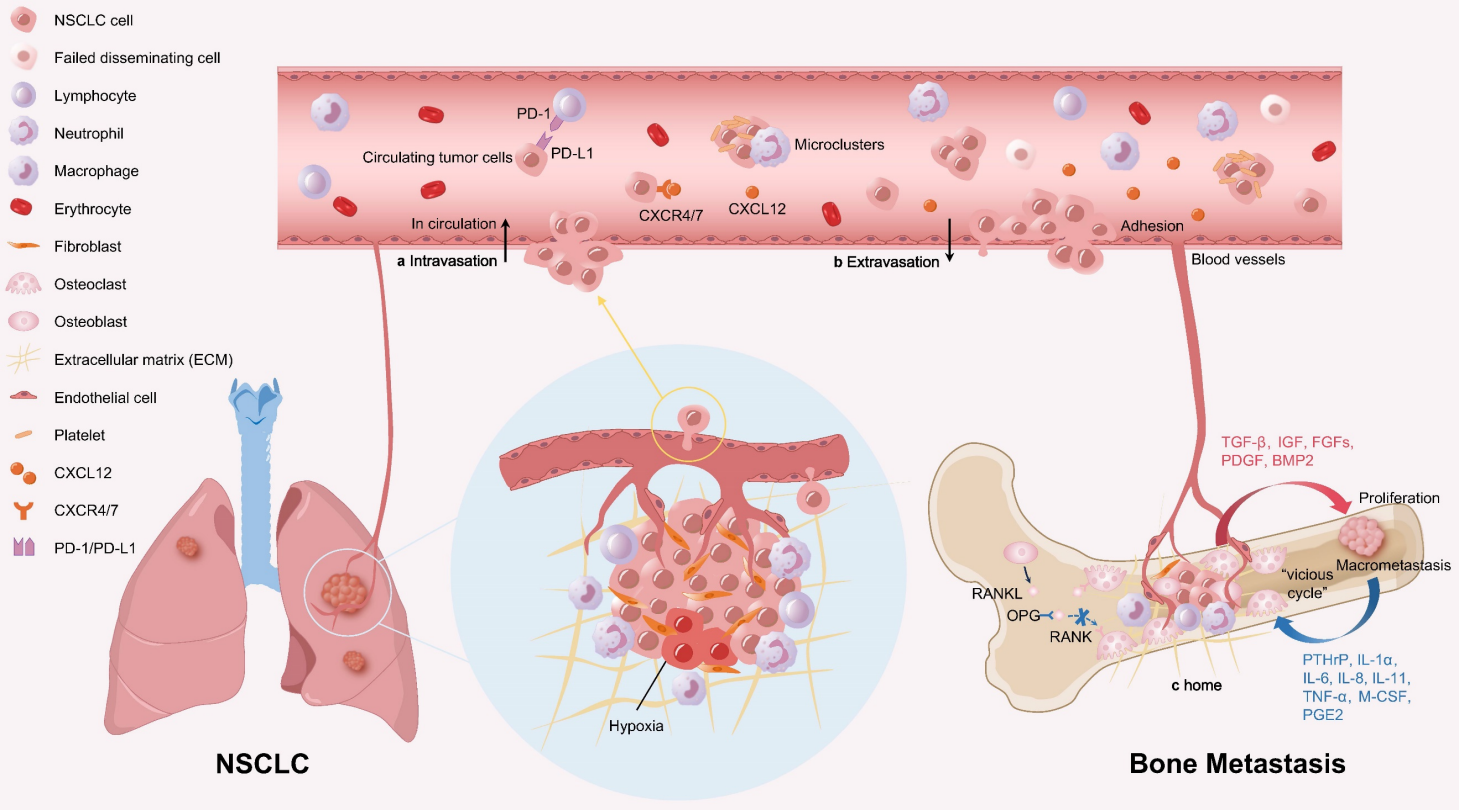
** Stepwise progression of bone metastasis in NSCLC.** a. NSCLC cells detach from the primary site and invade the circulation. In circulation, CTCs circulate as single cells or in microclusters coated with platelets, neutrophils, and other cells, making them difficult to eliminate. CTCs also express PD-L1 to evade the clearance of immune cells. b. CTCs adhere to the endothelium of bone blood vessels, then extravasate into bone to seed new metastasis. Various chemokines and adhesion molecules participate in CTCs extravasation. For example, NSCLC cells overexpressing CXCR4 are attracted to CXCL12 expressed in the bone matrix. c. NSCLC cells colonize in bone tissue and form macrometastases. Interactions between the NSCLC cells and the bone microenvironment enhance bone remodeling and tumor growth. Furthermore, osteolytic bone resorption and tumor cell proliferation form a "vicious cycle" that promotes bone metastasis.

Bone is a highly vascularized organ, especially the axial bone such as spine and ribs. The trabecular bone in these sites is closely adjacent to sinusoidal blood vessels, which are rich in blood supply and slow in blood flow, facilitating the adhesion and colonization of NSCLC cells on the sinusoidal endosteum, thereby promoting the occurrence of bone metastasis. Bone metastasis of NSCLC is a multistep process, including three stages: A. Detachment from the primary site and intravasation into the circulation; B. Extravasation into the bone microenvironment; C. Colonization in bone tissue and formation of metastatic tumors (Figure 1).

## Process of Non-Small Cell Lung Cancer Bone Metastasis

### 1. Detachment from the primary site and invasion into the circulation

Epithelial-mesenchymal transition (EMT) is a biological process in which epithelial cells undergo a specific program to transform into cells with mesenchymal phenotypes, characterized by reduced intercellular adhesion and increased motility and migration ability. Studies have shown that EMT is a key process for NSCLC cells to acquire invasive and metastatic phenotypes and is regulated by various factors^12^. Lung adenocarcinoma (LUAD) is the most common type of NSCLC, characterized by its high tendency to relapse and metastasize due to EMT of cancer cells^13^, ^14^. According to statistics, bone metastasis occurs most frequently in LUAD among all NSCLC types^5^. Wang *et al.*^15^ established a nude mouse model of multiorgan metastasis of LUAD and screened out lung metastasis and bone metastasis cell subtypes. Compared to lung metastasis cells, bone metastasis cells exhibited greater stemness and EMT plasticity. Mechanistic studies indicate that bone metastasis cells can activate macrophages to produce a high level of transforming growth factor (TGF)-β1, thereby promoting EMT and invasion of LUAD cells via TGF-β/SMAD2/3 signaling pathway. Preferentially expressed antigen of melanoma (PRAME), which is downregulated in LUAD and lung bone metastasis, plays a protective role in preventing bone metastasis of NSCLC cells. Knockdown of PRAME promotes EMT by decreasing the expression of E-cadherin and significantly increases bone metastasis *in vivo*^16^. Fas apoptotic inhibitory molecule 2 (FAIM2) is highly expressed in NSCLC tissues with bone metastasis and can promote NSCLC cell growth and bone metastasis by regulating the EMT process and the Wnt/β-catenin signaling pathway^17^. Knockdown of the proto-oncogene GNAQ in lung cancer cells also promotes bone metastasis by inducing EMT^18^. In addition, exosome-mediated delivery of miRNAs can promote metastasis of lung cancer cells and EMT by activating the STAT3 signaling pathway and Hippo pathway^19^, ^20^.

Hypoxia is a common characteristic of solid tumors, which enhances the malignant behavior of NSCLC cells. On the one hand, uncontrolled proliferation of malignant cells leads to increased oxygen consumption, while the existing vasculature cannot support the malignant cells' rapid proliferation and their demands for nutrients and oxygen. On the other hand, the new blood vessels stimulated by the tumor are poorly organized and structurally abnormal^21^, ^22^. Consequently, hypoxic areas are commonly present in tumors. Hypoxia-inducible factor-1α (HIF-1α), produced in the hypoxic tumor microenvironment (TME), is closely associated with tumor metastasis. A retrospective analysis indicates that HIF-1α is highly expressed in NSCLC and can be considered a predictive factor of bone spread and poor prognosis^23^. Bone marrow-derived mesenchymal stem cells (BMSCs) are components of the TME that can promote cancer progression. Exosomes derived from hypoxic BMSCs contain miRNAs (miR-193a-3p, miR-210-3p and miR-5100), which enhance the expression of markers associated with EMT by activating the STAT3 signaling pathway, thereby promoting the invasion of lung cancer cells^19^. Furthermore, hypoxic BMSC-derived extracellular vesicles can deliver miR-328-3p to lung cancer cells, and miR-328-3p can enhance the proliferation, invasion, migration, and EMT of these cells through the NF2-mediated Hippo pathway^20^.

NSCLC cells can secrete or induce stromal cells to produce matrix metalloproteinases (MMPs) to degrade the extracellular matrix (ECM), creating local defects in the basement membrane, which aids NSCLC cells in passing through and invading the stroma. MMP-1, MMP-2, MMP-7, MMP-19, and others are associated with NSCLC metastasis^24^. Bone sialoprotein (BSP) promotes lung cancer bone metastasis by inducing its downstream target gene MMP14^25^. NSCLC cells that enter the stroma invade the lymphatic system or blood vessels to become circulating tumor cells (CTCs). CTCs can circulate in microclusters coated with platelets, neutrophils, and other cells, making them difficult to eliminate^26^. CTCs can also express programmed cell death 1 ligand 1 (PD-L1) to evade immune cells clearance^27^. However, most CTCs are eliminated by physical, biochemical, and immunological stressors, with only a few surviving.

### 2. Extravasation into the bone microenvironment

The extravasation of CTCs requires the involvement of chemokines and adhesion molecules. Chemokines, especially C-X-C motif chemokine ligand 12 (CXCL12) and its receptor C-X-C receptor (CXCR) 4, play a crucial role in the homing of NSCLC cells from circulation to the bone. NSCLC cells overexpressing CXCR4 are attracted to CXCL12 expressed in the bone matrix^28^. CXCR4 is highly expressed in the bone destruction area of metastatic NSCLC samples and is associated with poor survival in NSCLC patients with bone metastases. CXCR4 can promote NSCLC-induced osteoclast differentiation and increases osteoclast formation. Overexpressed CXCR4 also promotes NSCLC to secrete soluble vascular cell adhesion molecule-1 (sVCAM1), which further increases osteoclastogenesis^29^. Apart from CXCR4, CXCR7 has also been identified as a new receptor for CXCL12. Recent studies indicate that CXCR7 is highly expressed in LUAD tissues. Overexpression of CXCR7 enhances the migration and polarization of lung cancer cells *in vitro*. In a xenograft model, CXCR7 promotes the growth of primary lung tumors and their metastases to secondary organs, such as the liver or bone marrow^30^. The CXCR7 agonist VUF11207 can bind to CXCR7, inhibiting CXCL12-mediated osteoclastogenesis and bone resorption^31^.

CTCs adhere to the endothelium of bone blood vessels, then penetrate through the endothelium and basement membrane to form new metastases. Adhesion molecules, including the integrin family, the selectin family and the cadherin family, have been shown to be closely associated with tumor cell adhesion. Intercellular adhesion molecule-1 (ICAM-1) is highly expressed in vertebral bone and serves as a downstream effector of C-X3-C motif chemokine ligand 1 (CX3CL1) signaling in vertebral bone marrow endothelial cells (VBEMCs). Overexpression of ICAM-1 can bind to the receptor lymphocyte function-associated antigen-1 (LFA-1) on NSCLC cells. Further research suggests that VBEMCs can coordinate with platelets through the CX3CL1/ICAM-1/LFA-1 pathway to functionally induce the adhesion of circulating NSCLC cells to the vertebral microvascular endothelium. In addition, CX3CL1/ICAM-1 can enhance the permeability of VBMECs through the Src/GEF-H1 pathway and induce transendothelial migration of NSCLC cells. Taken together, the CX3CL1/ICAM-1 signaling network between NSCLC cells and VBEMCs may be a potential novel target for preventing NSCLC spinal metastasis^32^. Moreover, the adhesion molecule CD24 is highly expressed in bone-directional lung cancer cells and promotes bone metastasis both *in vitro* and *in vivo* by promoting anchorage-independent growth and adhesion^33^.

### 3. Colonization in bone tissue and formation of metastatic tumors

#### 3.1 Tumor cell dormancy in bone metastasis

After extravasation from the vasculature, CTCs disseminate into the bone tissue as disseminated tumor cells (DTCs). However, DTCs face considerable barriers to colonization and growth in the bone microenvironment. Previous studies have demonstrated that DTCs preferentially locate to the hematopoietic stem cells (HSCs) niche of the bone and compete for survival space with the normal cellular occupants of the niche^34^. Furthermore, the bone marrow is a predominant site for immune cells, where some DTCs are eliminated by immune cells, including macrophages, natural killer (NK) cells, and infiltrating T cells. Surviving DTCs may be delivered to different locations within the bone endosteum^35^. Notably, approximately 80% of the endosteum bone surface is quiescent, with only a minority undergoing active bone remodeling. Bone remodeling occurs in the bone remodeling compartments (BRCs), which are rich in factors conducive to growth and survival. Tumor cells delivered to the BRCs may proliferate immediately, whereas those arriving at quiescent endosteal surfaces are more likely to become dormant^36^. Within the bone, dormant tumor cells can remain quiescent for years or even decades before re-initiating proliferation and causing overt metastasis.

Evidence has illustrated that dormancy is a reversible state, with osteoblasts and osteoclasts regulating the switch between dormancy and proliferation in DTCs^37^. Osteoblasts initiate and maintain tumor cell dormancy through interactions with tumor cells^38^. Conversely, osteoclasts can release dormant cells from the microenvironment by remodeling the bone to remove bone lining cells^37^. Dormant tumor cells are reactivated and begin to proliferate, leading to metastatic growth. Osteocytes also play a role in tumor dormancy. When mechanically stimulated, osteocytes release small extracellular vesicles containing miR-99b-3p, which directly target MDM2 to inhibit NSCLC cell proliferation and maintain dormancy^39^.

Hypoxia is another critical factor regulating tumor dormancy. Despite very high vascular density in bone marrow, the overall level of oxygenation is low, making it a hypoxic environment^40^. Current studies have found that regional oxygen tension in bone is heterogeneous, primarily controlled by the level of cellularity, oxygen consumption rate and oxygenated blood supply^41^. The leukaemia inhibitory factor receptor (LIFR) is a regulator of breast cancer dormancy. In breast cancer patients, LIFR expression is downregulated in those with bone metastases and is significantly negatively correlated with hypoxic gene activity. The hypoxic bone marrow environment reduces the LIFR/STAT3/SOCS3 signaling pathway in breast cancer cells. Loss of the LIFR or STAT3 enables dormant breast cancer cells to downregulate dormancy genes, and then proliferate in and colonize the bone^42^. Besides hypoxia, the overexpression of parathyroid hormone-related protein (PTHrP) secreted by bone DTCs can also inhibit LIFR expression. Studies have shown that histone deacetylase (HDAC) inhibitors can stimulate LIFR, even reversing the suppression of LIFR by hypoxia and PTHrP, thereby inducing a dormant phenotype in breast cancer cells^43^. This indicates that regardless of the mechanisms leading to LIFR downregulated in breast tumors and bone DTCs, HDAC inhibitors may still be used to induce tumor dormancy to reduce breast cancer recurrence and improve prognosis. In addition, the presence of tumors exacerbates the hypoxic bone microenvironment, affecting cellular metabolism and leading to excessive activation of osteoclasts, which disrupts the dynamic balance of bone remodeling^44^. Conversely, exposure to hyperbaric oxygen suppresses osteoclastogenesis and bone resorption *in vitro*^45^. Murphy *et al.*^46^ developed manganese dioxide (MnO_2_) nanoparticles that can provide sustained oxygenation to bone metastatic lesions, effectively reversing hypoxia. Their study shows that the sustained control of hypoxia in bone metastases enhances the cytotoxicity of NK cells against cancer spheroids, thereby increasing the survival rate of mice with bone metastases.

#### 3.2 Immune regulation in bone metastasis

Bone is typically immunoprivileged, facilitating the seeding and immune evasion of dormant tumor cells. It has been widely demonstrated that CD8+ T-cells, NK cells, and M1 macrophages can mediate anti-tumor responses in bone, whereas immunosuppressive cells such as M2 macrophages, myeloid-derived suppressor cells (MDSCs), and T- regulatory cells (Tregs) can attenuate the activity of CD8+ T-cells involved in tumor immune surveillance, thereby promoting the progression of bone metastasis^35^, ^47^. Immune checkpoint inhibitors (ICIs) targeting T-cells receptors cytotoxic T-lymphocyte-associated protein 4 (CTLA-4), programmed cell death 1 (PD-1), and its ligand PD-L1, aimed at increasing or activating immune cells to reverse the immunosuppressive state, are a promising therapeutic strategy for NSCLC bone metastasis^48^.

CD8+ T-cells, also known as cytotoxic lymphocytes (CTLs), play a crucial role in killing target cells and are considered the preferred immune cells against cancer. Arellano *et al.*^49^ found that non-activated T-cells infiltrating bone metastases express receptor activator of nuclear factor κB ligand (RANKL) and tumor necrosis factor-α (TNF-α), thereby increasing osteoclast formation and osteolytic metastasis in mice. Activated T-cells produce interferon-γ (IFN-γ) and interleukin (IL)-4, inhibiting osteoclast formation. This study suggests that immunotherapy aimed at activating T-cells may not only enhance their capacity to kill cancer cells but also prevent osteoclast formation and inhibit bone resorption. Transcriptional analysis of bone marrow cells shows that PD-L1 deficiency upregulates immune-stimulatory genes, promotes macrophage polarization to M1, enhances IFN-γ signaling, and increases the recruitment and activation of CTLs, thereby enhancing the anti-tumor immunity of the TME^50^. The study also found that PD-L1 deficiency in the hematopoietic or myeloid lineage can inhibit osteoclast differentiation and reduce bone metastasis.

NK cells not only kill tumor cells non-specifically through direct cytotoxic effects but also produce pro-inflammatory cytokines such as IFN-γ to inhibit tumor cell proliferation. However, the activity of NK cells can be evaded by the TME. Tumor cells in the immunosuppressive TME secrete immunosuppressive factors such as TGF-β, prostaglandin E2 (PGE2), and IL-10 that downregulate the activation receptors of NK cells, thus preventing their activation^51^. Preclinical studies have shown that adapter chimeric antigen receptor (AdCAR)-engineered NK-92 cells combined with therapeutic antibodies can target antigens expressed by different tumor bone metastasis cell lines and eliminate bone metastasis cells *in vitro*, demonstrating specific cytotoxic potential^52^.

Macrophages can be categorized into pro-inflammatory M1 macrophages and immunosuppressive M2 macrophages^53^. M2 macrophages secrete inhibitory cytokines such as IL-10 and TGF-β to downregulate immune responses, which has been proven to promote tumor growth and metastasis^54^. Recent studies have found that LUAD bone metastasis cells carrying the CD74-ROS1 fusion can induce M2 polarization of macrophages via the activation of the STAT3/CCL5 axis. M2 macrophages produce high levels of TGF-β1, promoting LUAD cell invasion through TGF-β/SMAD2/3 signaling. *In vivo*, targeting the CD74-ROS1/STAT3/CCL5 axis with Crizotinib (a ROS1 inhibitor) and Maraviroc (a CCL5 receptor inhibitor) strongly inhibits bone metastasis and secondary metastasis of LUAD bone metastasis cells, providing an alternative strategy for the treatment of LUAD bone metastasis^15^.

MDSCs can suppress anti-tumor immune responses through various mechanisms and promote tumor progression and metastasis to distant sites, including bone^55^, ^56^. Exosomes produced by MDSCs can overly activate or deplete CD8+ T-cells, thus suppressing immune functions^57^. In NSCLC patients treated with PD-1 immunotherapy, lower levels of MDSCs are associated with longer progression-free survival (PFS) and OS^58^. Furthermore, MDSCs can differentiate into osteoclasts, inducing bone resorption in mouse models of breast cancer bone metastasis^59^. Therefore, targeting MDSCs may be an effective strategy to reduce osteolytic metastasis. However, another study indicates that eliminating MDSCs alone with anti-Gr1 does not affect the growth of bone metastatic tumors. In contrast, the combined use of anti-Gr1 with bone-targeting drugs [zoledronic acid (ZA)] significantly reduces the growth of established bone metastatic tumors^60^.

Tregs are known for their immunosuppressive capacity and are often involved in tumor initiation and progression^61^. An increase in Tregs in the bone marrow has been observed in prostate cancer patients with bone metastasis. Mechanistic studies have found that active Tregs migrate to the bone marrow through the CXCR4/CXCL12 signaling pathway and proliferate there. In mouse breast cancer bone metastases, using the CXCR4 antagonist AMD3465 combined with the indoleamine 2, 3-dioxygenase 1 (IDO1) inhibitor D-1-methyltryptophan to block the CXCL12/CXCR4 axis reduces the number of Tregs within the tumor and weakens immune suppression, thus inhibiting the progression of breast cancer bone metastasis and prolonging mouse survival^62^. In addition to their immunosuppressive function, Tregs can inhibit osteoclast differentiation and promote bone formation in mice with prostate cancer, leading to osteoblastic metastasis^63^. However, depletion of Tregs *in vivo* leads to reduced bone density in mice with prostate cancer. Therefore, targeting Tregs may not only enhance anti-tumor immunity but also improve bone pathology.

#### 3.3 Bone remodeling in bone metastasis

Normal bone metabolism is a homeostatic process involving the resorption of bone by osteoclasts and the formation of new bone by osteoblasts. NSCLC cell migration to the bone disrupts this balance and alters the bone microenvironment, promoting the development of bone metastasis. Bone metastases can be categorized into osteolytic, osteoblastic, and mixed types. Most NSCLC bone metastases are osteolytic, where osteoclast-mediated bone resorption predominates, characterized by bone loss and bone dissolution. The bone matrix is rich in various cell factors, including TGF-β, insulin-like growth factor 1 (IGF), fibroblast growth factors (FGFs), platelet-derived growth factor (PDGF), bone morphogenetic protein 2 (BMP2), and others. During osteolytic bone resorption, these factors are released and activated in the bone microenvironment, promoting the proliferation of tumor cells at the bone metastasis site^64^. Furthermore, tumor cells at bone metastasis sites release various cytokines, such as PTHrP, IL-1α, IL-6, IL-8, IL-11, TNF-α, macrophage colony-stimulating factor (M-CSF) and PGE2, which induce osteoclast formation and promote osteolytic bone resorption. This osteolytic bone resorption and tumor cell proliferation create a "vicious cycle" that further promotes bone metastasis^65^.

## Factors Regulating Non-Small Cell Lung Cancer Bone Metastasis

### 1. Signaling pathways

**Figure 2:**
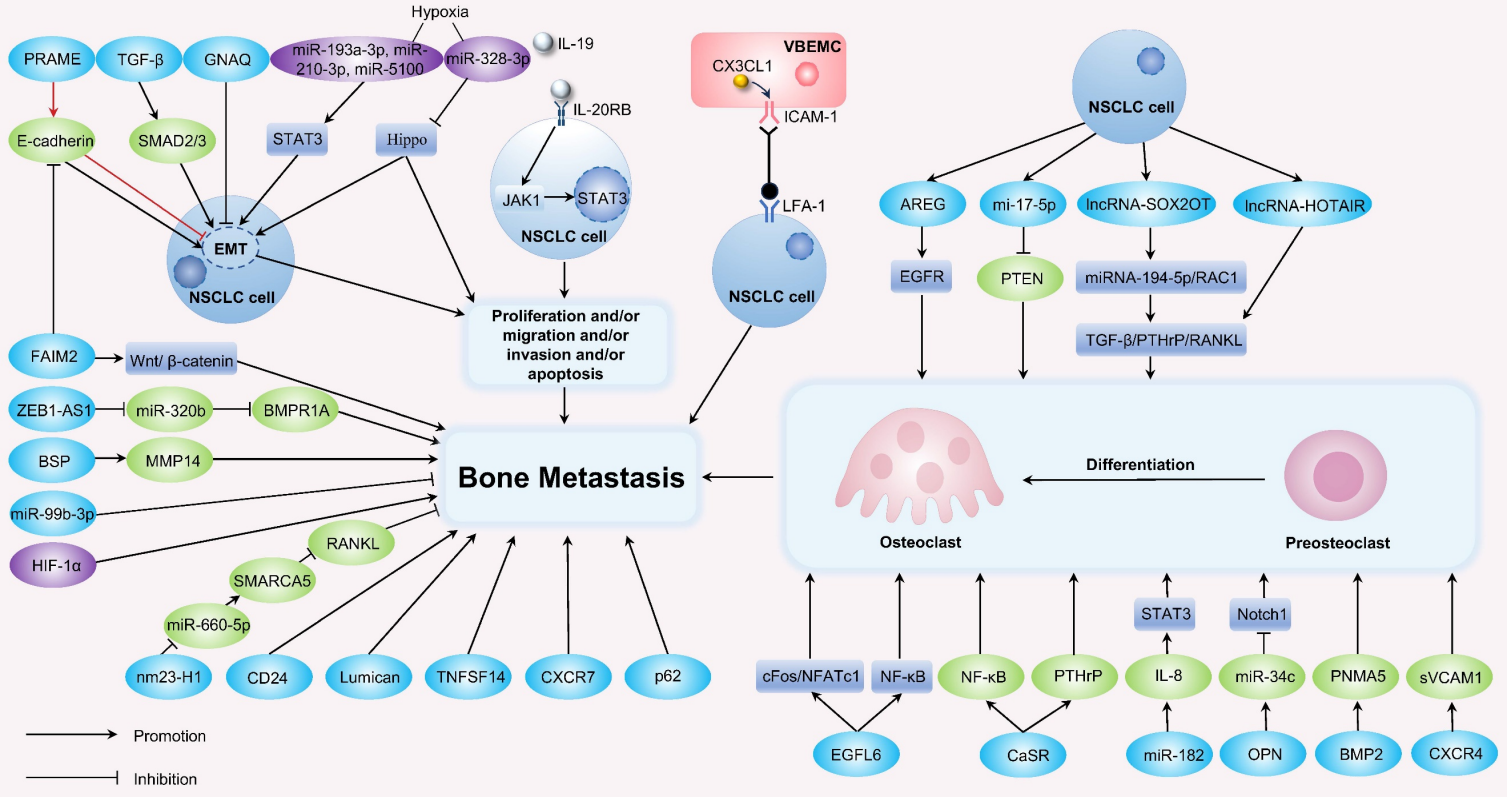
Main factors and signaling pathways contributing to bone metastasis in NSCLC.

Several mechanisms are involved in the regulation of bone metastasis (Figure 2, Table 1). The receptor activator of nuclear factor κB (RANK)/RANKL/OPG pathway is a key pathway that regulates osteoclast differentiation and bone remodeling, playing a significant role in osteolytic metastasis. RANK, expressed by osteoclasts, osteoclast precursors, and some tumor cells, can bind to RANKL expressed by bone marrow stromal cells, osteoblasts, and bone cells to stimulate osteoclast differentiation. Osteoprotegerin (OPG), produced by osteoblasts and bone cells, binds to RANKL to prevent the binding of RANK and RANKL, thereby inhibiting osteoclast activity and exerting a bone-protective effect^66^. In a study of 73 patients with metastatic NSCLC, the expression of RANKL and the RANKL/OPG ratio are significantly higher in patients with bone metastases than in those without^67^. Furthermore, the increase in the RANKL/OPG ratio is associated with an increased incidence of bone metastasis. In addition, the IL-19/IL-20RB/STAT3 axis plays a critical role in mediating the direct protumor effects of the osteoclast niche. Importantly, neutralizing antibodies that block IL-20RB can significantly inhibit lung cancer bone metastasis, demonstrating the potential for treating bone metastasis^68^. MiR-182 is significantly upregulated in bone-metastatic NSCLC cell lines and tumor specimens, serving as a key regulatory factor for bone metastasis of NSCLC cells. The miR-182/IL-8/STAT3 axis is an important signaling pathway in the regulation of osteoclast differentiation and osteolytic bone metastasis of lung cancer^69^. Osteopontin (OPN), an acidic protein secreted by stromal cells and cancer cells, is essential for maintaining bone homeostasis. High expression of OPN in NSCLC is closely associated with malignant progression and poorer survival outcomes in NSCLC patients. The deletion of OPN can inhibit osteolytic bone metastasis in NSCLC patients by regulating bone metabolism through miR-34c/Notch1 pathway^70^. The level of the exocrine protein epidermal growth factor-like domain multiple 6 (EGFL6) in LUAD tissues have been reported to correlate with bone metastasis in patients. In a nude mouse model, overexpression of EGFL6 could enhance tumor growth and result in greater bone destruction. *In vitro*, EGFL6 could promote osteoclast differentiation and bone resorption in mouse bone marrow mononuclear macrophages (BMMs) by activating the NF-κB and c-Fos/NFATc1 signaling pathways^71^. Additionally, lncRNA ZEB1-AS1 is significantly upregulated in NSCLC tissues, and its increased expression is related to poor OS for NSCLC patients^72^. Another study has found that lncRNA ZEB1-AS1 is expressed at higher levels in lung cancer bone metastasis tissues compared to lung cancer tissues. Both *in vitro* and *in vivo* experiments confirm that ZEB1-AS1 promotes bone metastasis by targeting the miR-320b/BMPR1A axis in LUAD^73^. Finally, the metastasis suppressor gene nm23-H1 has been shown to inhibit tumor progression and bone-specific metastasis of lung cancer by regulating the miR-660-5p/SMARCA5/RANKL axis^74^.

Exosomes are special extracellular vesicles. In recent years, accumulating evidence has highlighted the close association between exosomes and tumor metastasis. Exosomes precede tumor cells to metastatic sites and alter the microenvironment to create a favorable milieu for the attachment and proliferation of CTCs^75^. In NSCLC patients with bone metastasis, exosomes in peripheral blood are highly enriched with lncRNA-SOX2OT, whose high expression is associated with shorter OS^76^. Mechanistically, lncRNA-SOX2OT regulates osteoclast differentiation and stimulates bone metastasis by targeting the miRNA-194-5p/RAC1 signaling axis as well as TGF-β/PTHrP/RANKL signaling pathway in osteoclasts. Thus, lncRNA-SOX2OT can serve as a prognostic biomarker for NSCLC patients with bone metastasis. Exosomes lncRNA HOTAIR from NSCLC also promotes bone resorption by targeting the TGF-β/PTHrP/RANKL signaling pathway^77^. Amphiregulin (AREG) contained in NSCLC exosomes can activate the EGFR pathway in pre-osteoclasts, inducing their differentiation and consequently promoting osteolytic bone metastasis^78^. NSCLC exosomal miR-17-5p promotes osteoclastogenesis through the PI3K/Akt pathway via targeting PTEN^79^. Exosomes can be detected in various biological fluids such as serum, saliva, and urine. Numerous miRNAs within plasma exosomes exhibit remarkable stability in bodily fluids, making them ideal candidates for clinical detection as well as biomarkers for cancer detection and prognosis prediction^80^. Additionally, studies suggest that combining exosomal RNA and circulating tumor DNA (ctDNA) can increase the sensitivity of detecting EGFR mutations in the plasma of NSCLC patients, particularly when ctDNA levels are low^81^. Circulating miR-141 and miR-375 have been identified as biomarkers for diagnosis and prognosis of bone metastasis in prostate cancer^82^. Meta-analyses have confirmed the high diagnostic value of plasma exosomal miRNAs in patients with prostate or breast cancer^83^, ^84^. Bioinformatic analysis also suggests that exosomes hsa-miR-151a-3p and hsa-miR-877-5p may serve as novel biomarkers for predicting bone metastasis in NSCLC^85^. Overall, certain exosomal miRNAs can serve as biomarkers for tumor bone metastasis, but further research is needed to screen and validate miRNAs associated with NSCLC bone metastasis.

### 2. Other factors

BMP2 has been reported to be highly expressed in NSCLC, and activation of BMP2 signaling can promote the proliferation, migration, invasion, and metastasis of LUAD cells^86^. Furthermore, BMP2 can induce osteoclast differentiation and promote bone metastasis through its direct downstream target gene PNMA5^87^. Lumican, a major proteoglycan component of the bone matrix, is mainly expressed in differentiated and mature osteoblasts. Lumican promotes lung cancer cell metastasis to bone via an autocrine regulatory mechanism^88^. Liu *et al.*^89^ used immunohistochemistry (IHC) to detect the expression level of calcium-sensing receptor (CaSR) in 120 cases of LUAD bone metastasis tissues, finding that the expression of CaSR in bone metastatic lung cancer tissues was significantly higher than that in non-metastatic lung cancer tissues. Further research showed that CaSR can positively regulate the expression of NF-κB and PTHrP in NSCLC cells, thereby promoting osteoclast maturation and the development of bone metastasis in NSCLC. Overexpression of autophagy protein p62 is associated with poor prognosis in patients with LUAD bone metastasis, and its excessive expression can promote bone metastasis of LUAD^90^. Tumor necrosis factor superfamily member 14 (TNFSF14) has also been shown to promote osteolytic bone metastasis in patients with NSCLC^91^.

In summary, multiple mechanisms contribute to the occurrence of bone metastasis in NSCLC. Various signaling pathways and factors participate in the metabolic crosstalk between tumor cells and the bone microenvironment. Among these, exosomes are a special form of participation in the crosstalk. Exosomes are capable of transporting various DNAs, RNAs, and proteins between tumor cells and adjacent cells, thus participating in the regulation of bone metastasis. Many exosomal miRNAs are biologically stable in body fluids and blood circulation, and can function as biomarkers for the diagnosis of bone metastasis.

## Clinical Treatment of Bone Metastasis

Currently, the treatment of bone metastasis in NSCLC includes treatment of the primary and metastatic sites. Although appropriate systemic treatment should be chosen for patients with stage IV NSCLC, those with small burden of metastasis may benefit from aggressive local treatment to the primary tumor. Multiple studies have demonstrated that surgical resection or radiotherapy of the primary site is beneficial for the survival of patients with oligometastatic NSCLC^92^-^94^. Treatment for bone metastatic sites mainly includes surgery, radiotherapy, BTAs, chemotherapy, targeted therapy, and immunotherapy (Table 2).

### 1. Surgery

NSCLC bone metastasis can lead to decreased bone strength, and once pathological fractures occur, patients' QoL would be significantly compromised without appropriate surgical intervention. Surgery can maintain patients' limb function and mobility by preventing impending pathological fractures, stabilizing existing pathological fractures, controlling spinal cord compression, and alleviating pain. For patients with spinal metastases secondary to NSCLC, a meta-analysis shows that patients who undergo surgical intervention experience significant pain relief, neurological function improvements, and some even regain mobility^95^. Another study has found a correlation between the duration of neurological deficit and survival time in NSCLC patients with spinal cord compression, suggesting that early treatment may lead to better prognosis^96^. Hershkovich *et al.*^97^ found that urgent surgery and radiotherapy resulted in greater functional improvements in patients with acute metastatic spinal cord compression compared to urgent radiotherapy alone. Although a consensus on the optimal treatment for NSCLC patients with spine metastases has yet to be established, surgical intervention demonstrates substantial benefits in relieving pain and improving nerve function. Furthermore, prophylactic internal fixation is associated with lower costs, shorter hospital stays, and better prognosis compared with fixation after fractures^8^, ^98^. Consequently, prophylactic surgery for impending fractures may be a cost-effective approach.

### 2. Radiotherapy

Radiotherapy is a relatively simple and effective method of treating bone metastasis from NSCLC, and has been proven effective in alleviating pain, preventing pathological fractures, and reducing the need for further surgery^99^. It's commonly employed to treat bone pain secondary to various cancers, with an overall response rate of about 70% and a complete response rate of 30%^100^. Radiotherapy includes external beam radiotherapy (EBRT) and radionuclide therapy.

#### 2.1 External beam radiotherapy

EBRT remains the preferred treatment for moderate to severe bone pain caused by bone metastasis. A prospective study showed that previously unirradiated patients with painful bone metastasis experienced significant pain relief and improved QoL after radiotherapy^101^. A study by Shulman *et al.* found that EBRT reduced the risk of pain and SREs in patients with asymptomatic bone metastases, but did not improve OS^102^. This suggests that prophylactic radiotherapy may also be beneficial in delaying the occurrence of pain and reducing the incidence of SREs in patients with asymptomatic bone metastases. In recent years, a novel radiotherapy technique, stereotactic body radiotherapy (SBRT), has been shown not only to increase the biological dose effect but also to reduce damage to healthy tissues. Several studies have indicated that patients with solid tumor bone metastases who received SBRT had a higher complete response rate for pain compared to conventional EBRT, suggesting a potential benefit of SBRT in reducing pain^103^-^105^. In addition to controlling local symptoms, in rare cases, radiotherapy can produce an abscopal effect, where tumors outside the irradiated area also show shrinkage, opening up new avenues for research in radiotherapy^106^.

However, radiotherapy for bone metastasis may cause clinically significant hematological toxicity. The bone marrow consists of two types of multipotent stem cells: hematopoietic stem cells and mesenchymal stem cells. Hematopoietic stem cells produce myeloid and lymphoid lineages. Lymphocytes are among the most radiosensitive cells, showing an immediate decrease after irradiation and reaching a low point within 1-2 months of starting treatment.

This toxicity results from the direct effects of radiation on circulating lymphocytes and the indirect effects on bone marrow stem cells^107^. However, bone marrow mesenchymal stem cells can retain their stem cell properties even after exposure to high doses of radiation, demonstrating a relative resistance to ionizing radiation. The mechanisms underlying this radioresistance may involve efficient DNA damage recognition, double strand break repair and evasion of apoptosis^108^.

#### 2.2 Radionuclide therapy

Radionuclide therapy is an alternative treatment option for patients with widespread bone metastases confirmed by ^99^Tcm-MDP bone scintigraphy to have localized accumulations at the metastatic lesions. Strontium-89 (^89^Sr) is the most commonly used radiopharmaceutical agent in internal radiotherapy for bone metastases, which can relieve bone pain^109^, ^110^. However, a meta-analysis of bone metastasis in NSCLC patients found that ^89^Sr had no statistically significant impact on OS or SREs^111^. A recent study found that injection of ^89^Sr relieved pain in patients with painful bone metastasis at previously irradiated site without causing grade 3 or worse adverse events (AEs)^112^. This suggests that ^89^Sr may be an option for the treatment of painful bone metastasis in patients with a history of prior irradiation.

### 3. Bone-targeted agents

#### 3.1 Bisphosphonates

BPs are fundamental medications for treating bone metastasis and are used in combination with conventional anti-tumor therapy, becoming a part of multidisciplinary treatment of NSCLC patients with bone metastases. A retrospective study involving 359 lung cancer patients with bone metastases treated with BPs indicated that prolonged use of BPs (>24 months) may increase the risk of AEs such as renal impairment. However, compared with patients treated for ≤24 months, those who continued treatment beyond 24 months experienced prolonged early survival without SREs. Patients may receive continuous BPs therapy beyond 24 months to prolong SRE-free survival, but close monitoring for potential AEs is necessary^113^. ZA is a third-generation bisphosphonate that has been approved by the Food and Drug Administration (FDA) for the treatment of bone metastasis in various cancers. It inhibits the synthesis of structural proteins in osteoclasts, induces osteoclast apoptosis, reduces osteoclast-mediated bone resorption, and significantly lower hypercalcemia resulting from excessive bone resorption. A meta-analysis showed that compared with the control group, NSCLC patients with bone metastases who received ZA had a reduced incidence of SREs and benefited in terms of OS^111^.

It's important to be aware of the potential adverse effects of BPs, such as osteonecrosis of the jaw (ONJ), renal toxicity, and hypocalcemia, and early aggressive treatment should be taken if these occur^114^. The impaired osteogenic differentiation of orofacial BMSCs is a characteristic of bisphosphonate-related osteonecrosis of jaw (BRONJ). Corin is a key regulatory factor in skeletal development and orthopedic diseases. Recent studies have shown that METTL7A can reverse BPs-impaired BMSC function by modulating corin m6A modification, thereby promoting osteoblast differentiation, providing a new therapeutic option for BRONJ^115^. Additionally, local transplantation of adipose-derived stem cells (ADSCs) can revive bone remodeling and prevent BRONJ^116^. In BRONJ-like animal model, ADSCs transplantation can release TGF-β1, which activates osteoclastogenesis and rescues BMSC migration, effectively promoting bone healing of BRONJ.

#### 3.2 Denosumab

Denosumab is a monoclonal antibody that binds tightly to RANKL, preventing its interaction with the receptor RANK and inhibiting osteoclast-mediated bone resorption. In 2020, Denosumab was approved by the National Medical Products Administration (NMPA) for the treatment of SREs caused by bone metastases from solid tumors and multiple myeloma. Compared to BPs, denosumab has no renal toxicity, making it a safer option for patients with renal impairment. A multi-institutional retrospective cohort study has demonstrated that denosumab-induced ONJ is less likely than BP-induced ONJ^117^. Bozzo *et al.*^118^ conducted a network meta-analysis of 131 randomized controlled trials and found that patients treated with denosumab had better outcomes in terms of OS and time to SREs compared with those treated with ZA, and both were superior to untreated patients. There was no difference between patients treated with denosumab and ZA in terms of reducing the incidence of SREs. Additionally, discontinuation of denosumab may lead to rebound osteolysis and an increased risk of fractures^119^. Unlike BPs, which bind to the bone matrix and remain active for many years, denosumab does not bind to the bone matrix, so its inhibition of bone resorption ceases after discontinuing. Dupont *et al.*^120^ reported a case of a 43-year-old patient with LUAD and bone metastases who suffered four recent spontaneous rebound-associated vertebral fractures only 8 months after discontinuing denosumab. The ESMO Clinical Practice Guidelines recommend that BTAs should be initiated as soon as bone metastases are diagnosed and should be considered throughout the course of the disease^121^.

### 4. Chemotherapy

Platinum-based combination chemotherapy is the standard first-line treatment for stage IV NSCLC patients without driver alterations. Systemic chemotherapy can improve patients' general condition and enhance their QoL. For NSCLC patients with bone metastases, it is generally recommended to combine BTAs concurrently. Previous studies have shown that the combination of chemotherapy plus ZA can significantly reduce pain scores and the use of analgesic in NSCLC patients^122^. Peters *et al.*^123^ conducted a pooled analysis of the SPLENDOUR and AMGE-249 trials, revealing no statistically significant improvement in PFS/OS between the group receiving chemotherapy plus denosumab and the group receiving chemotherapy alone. This suggests that the addition of denosumab to standard first-line chemotherapy for advanced NSCLC does not provide significant benefits for patients in terms of PFS/OS.

### 5. Targeted therapy

Targeted therapy can alleviate tumor burden and significantly improve QoL for patients with oncogenic driver mutations^124^. Osimertinib, a third-generation EGFR-TKI, has been used for advanced EGFR mutation-positive NSCLC patients as monotherapy. Higuchi *et al.*^125^ established an EGFR-mutant LUAD bone metastasis mouse model and found that compared to mice not receiving osimertinib treatment, mice receiving osimertinib treatment showed tumor regression, prolonged mouse survival and bone remodeling in the bone metastasis models. Furthermore, three patients with advanced EGFR19del mutation LUAD and bone metastases had a relatively longer stable period of bone metastases (12-22.7 months) after receiving osimertinib treatment^126^. These findings suggest that osimertinib is a clinical option for EGFR exon 19 deletion-mutated LUAD patients with bone metastases, although further clinical studies are needed to confirm its efficacy.

### 6. Immunotherapy

ICIs have provided a new treatment option for advanced NSCLC patients. Based on the significant survival benefits observed in KEYNOTE series clinical trials, pembrolizumab monotherapy has been approved as a first-line treatment for NSCLC patients with PD-L1 positive^127^, ^128^. Qiang *et al.*^129^ conducted a retrospective study of 110 NSCLC patients with bone metastases and found that patients receiving first-line pembrolizumab therapy had higher objective response rates (ORR), longer PFS, and OS compared to those receiving second-line therapy or beyond. Both univariate and multivariate analyses indicated that first-line immunotherapy was an independent predictor of PFS and OS. These results suggest that advanced NSCLC patients with bone metastases should receive early immunotherapy to improve efficacy and prolong survival. Several studies have demonstrated that BTAs have synergistic effects when used in combination with ICIs, improving patient survival rates without increasing toxicity^130^-^132^. Furthermore, radioimmunotherapy shows advantages in clinical treatment of cancer and metastasis. Radiotherapy exhibits synergistic antitumor efficacy when used concomitantly with ICIs in patients with advanced NSCLC carrying bone metastases, which can alleviate symptoms and improve patients' survival^129^, ^133^. In addition to combination with BTAs or radiotherapy, ICIs can also be combined with anti-angiogenic agents. A retrospective study involving 95 NSCLC patients with bone metastases showed that the combination of ICIs and anti-angiogenic agents significantly improved PFS for bone metastases and potentially decreased SREs^134^.

### 7. Other

In recent years, whole-genome sequencing has revealed the importance of epigenetic alterations in bone remodeling and metastasis^135^-^137^. Epigenetic therapy, either as a single treatment or in combination with other existing therapies, has shown convincing results in enhancing anti-tumor effects and overcoming resistance^138^. HDACs are abnormally expressed in various cancers and can alter the expression of genes that drive tumor occurrence and metastasis^139^. Since HDAC inhibitors can cause bone loss, preclinical and clinical studies suggest that combining HDAC inhibitors with BTAs may be successful in treating bone metastatic diseases^140^. Unfortunately, due to the varied, sometimes even opposing effects of HDAC in different tumor types, the efficacy of HDAC inhibitors in different tumor types can vary significantly, so trials in multiple tumor types are needed. Research indicates that low-dose adjunctive epigenetic therapy can disrupt the premetastatic TME and inhibit metastasis, potentially serving as an adjuvant approach for cancer treatment^141^.

The mLPR is an mPLA/mRNA tumor vaccine that inhibits lung cancer progression and reduces bone metastases through the activation of immune cells, secretion of IFN-γ/IL-12 cytokines, and antibody dependent cellular cytotoxicity mediated by NK cells, providing new prospects for the treatment of lung cancer and bone metastasis^142^. Advances in nanotechnology also offer a novel strategy for the treatment of bone metastasis. Nanocarrier drug delivery systems can precisely deliver drugs to bone metastatic lesions while minimizing damage to healthy cells or organs, demonstrating significant advantages over traditional drug delivery methods^143^.

## Conclusion

In summary, bone metastasis is a common complication in NSCLC patients and is difficult to cure. It not only adversely affects QoL of NSCLC patients but also worsens their prognosis. Despite advances in research into the mechanisms and treatment of bone metastasis in NSCLC, metastatic bone disease remains a significant cause of NSCLC-related mortality. In this review, we first summarize the steps of NSCLC cells detaching from the primary site and migrating to the bone. Secondly, we explore the factors involved in the regulation of bone metastasis. Various signaling pathways and factors play crucial roles in metabolic crosstalk between NSCLC cells and the bone microenvironment, contributing to the development of bone metastasis.

Lastly, we review the current treatment options for NSCLC patients with bone metastasis. Overall, a multidisciplinary comprehensive treatment strategy should be adopted for patients. Beyond standardized systemic therapies, aggressive local treatment, such as surgery and radiotherapy, should be employed to maximize survival benefits, especially in oligometastatic patients. Additionally, several preclinical and clinical studies indicate that moderate exercise not only inhibits bone metastasis progression but also improves functional capabilities in patients with bone metastasis^39^, ^144^-^146^. However, research specifically focusing on NSCLC patients is limited, and further studies are necessary to establish the value of exercise in this population. Currently, agents targeting new therapeutic targets, including TGF-β, integrins, CXCR4, and PTHrP, are gradually entering clinical trials. The prognosis for NSCLC patients with bone metastasis remains poor, and further research is needed to develop novel bone-targeted drugs and to find better treatment strategies.

## Figures and Tables

**Table 1:** Factors contributing to bone metastasis of NSCLC

Factor	Expression	Clinical significance	Function	Mechanism(s)	Reference
TGF-β	Up	Bone metastasis	Migration, Invasion, EMT	Promoting TGF-β/SMAD2/3 signaling pathway	15
PRAME	Down	Bone metastasis	Proliferation, Migration, Invasion, EMT	Regulating E-cadherin signaling pathway	16
FAIM2	Up	Tumor stage, Lymph node metastasis, Bone metastasis, Poor prognosis	Proliferation, Migration, Invasion, Apoptosis, EMT	Promoting the EMT process and Wnt/β-catenin signaling pathway	17
GNAQ	Mutation	Metastasis, Drug resistance	Proliferation, Invasion, EMT	Promoting EMT and cancer stem cell (CSC)-like properties	18
miR-193a-3p, miR-210-3p, miR-5100	Up	Biomarkers for cancer metastasis	Migration, Invasion, EMT	Activating STAT3 signaling-induced EMT	19
miR-328-3p	Up	Cancer progression	Proliferation, Migration, Invasion, EMT	Inhibiting NF2-mediated Hippo axis	20
BSP	Up	Decreased OS, Advanced clinical disease stage, Bone metastasis	Migration, Invasion	Promoting MMP14	25
CXCR4	Up	Poor survival, Bone metastasis	Proliferation, Migration, Invasion	Self-potentiation and promoting VCAM1 secretion	29
CXCR7	Up	Tumor growth, Metastasis	Migration, Invasion and Polarization	No description	30
CX3CL1/ICAM-1	Up	Spinal metastasis	Migration, Invasion, Adhesion	Activating CX3CL1/ICAM-1/LFA-1 pathway and Src/GEF-H1 pathway	32
CD24	Up	Bone metastasis	No description	No description	33
miR-99b-3p	Up	Bone metastasis	Proliferation	Inhibiting the expression of MDM2	39
RANKL	Up	Tumor stage, Lymph node metastasis, Distant metastasis, Bone metastasis	Migration, Invasion	Regulating RANK/RANKL/OPG axis	66, 67
IL-20RB	Up	Bone metastasis	Proliferation, Migration	Regulating IL-19/IL-20RB/STAT3 axis	68
miR-182	Up	Bone metastasis	Migration	Promoting miR-182/IL-8/STAT3 axis	69
OPN	Up	Shorter OS, Shorter disease-free survival (DFS), Bone metastasis	Proliferation	Regulating miR-34c/ Notch1 pathway	70
EGFL6	Up	TNM stage, Bone metastasis	Proliferation, Migration, Invasion	Activating NF-κB and c-Fos/NFATc1 signaling pathways	71
lncRNA ZEB1-AS1	Up	Advanced TNM stage, Poor OS, Lymph node metastasis, Bone metastasis	Proliferation, Migration, Invasion, EMT	Regulating miR-320b/BMPR1A axis	72, 73
nm23-H1	Down	Bone metastasis	Proliferation, Migration, Invasion	Regulating miR-660-5p/SMARCA5/RANKL signaling pathway	74
lncRNA-SOX2OT	Up	Bone metastasis, Shorter OS	Migration, Invasion	Regulating miRNA-194-5p/RAC1 signaling axis and TGF-β/PTHrP/RANKL signaling pathway	76
lncRNA HOTAIR	Up	Bone metastasis	No description	Targeting TGF-β/PTHrP/RANKL pathway	77
AREG	Up	Poor prognosis, Bone metastasis	No description	Activating EGFR pathway	78
miR-17-5p	Up	Bone metastasis	No description	Inhibiting the expression of PTEN	79
BMP2	Up	Poor survival, Bone metastasis	Migration, Invasion	Inducing the expression of PNMA5	86, 87
Lumican	Up	Bone metastasis	Migration, Invasion, Adhesion	Through an autocrine regulatory mechanism	88
CaSR	Up	Bone metastasis	Proliferation, Migration	Promoting NF-κB and PTHrP expression	89
p62	Up	More bone lesions (>3), Shorter OS, Shorter PFS	Migration	Out of LC3-dependent autophagy	90
TNFSF14	Up	Bone metastasis	No description	No description	91

**Table 2:** Treatment of bone metastasis from solid tumors including NSCLC

Type of study	Treatment	Population	No. of Patients	Results	Conclusions	Reference
Retrospective	Radical treatment (surgery and/or chemotherapy and/or radiotherapy)	Patients with NSCLC and synchronous isolated bone metastases	41	The five-year OS, PFS, and DFS rates of patients who underwent pulmonary resection were 66.7%, 55.6%, and 44.4%, respectively. Primary lung tumor resection (HR = 4.18, 95% CI, 1.20-14.6, P = 0.025) and EGFR mutation (HR = 3.30, 95% CI, 1.08-10.1, P = 0.036) were significant predictors of OS in patients on multivariate analysis.	Patients with clinical N0-1 NSCLC and synchronous isolated bone metastases may achieve longer survival rates following primary lung tumor resection.	92
Retrospective	Surgery	Patients with bone synchronous oligometastatic NSCLC	27	Intraoperative and 30-days mortality was null. One major and 10 (37.04%) mild complications were recorded. 1-year and 5-years OS from the diagnosis and 1-year, 3-years DFS were 96%, 38%, and 66%, 30%, respectively.	Surgical treatment of primary NSCLC and bone synchronous metastasis seems to be safe and feasible and rewarding survivals may be expected.	93
Retrospective	Definitive vs Nondefinitive primary therapy	Patients with stage IV NSCLC and oligometastatic disease	53 vs 133	Definitive local therapy to the primary tumor was associated with prolonged survival (HR, 0.65, P = 0.043).	Definitive treatment to the primary tumor may be beneficial for the survival of patients with oligometastatic NSCLC.	94
Prospective	Surgery	Patients with metastatic spinal cord compression from NSCLC	50	An ECOG-PS improvement by at least one grade was observed in 66.0% of patients. The median overall survival (mOS) after surgery was 5.2 months. Major complications developed in 34.0% of patients, and the 30-day mortality rate was 10.0%.	Surgical treatment could improve functional outcomes as well as act as an adjuvant therapy to improve survival time by improving the functional status.	96
Retrospective	Urgent surgery and radiotherapy vs Urgent radiotherapy alone	Patients with acute metastatic spinal cord compression	32 vs 22	The Karnofsky functional score improved following surgery (p = 0.016), while it did not change significantly under radiotherapy (p = 0.466). The Karnofsky change following treatment was significantly better following surgery (p < 0.0001) while not improving under radiotherapy. 31.3% of patients regained sphincter function following surgery compared to 13.6% under radiotherapy (p < 0.0001). THE Kaplan-Meier survival curve analysis showed a trend toward better survival in the surgery group but did not reach statistical significance.	Urgent surgery and radiotherapy is superior to radiation treatment in treating acute metastatic spinal cord compression in all subgroups and early surgery improved function, motor strength, sphincter control, and ambulation without affecting life span.	97
Prospective	Radiotherapy	Patients with painful bone metastases	167	Reduced pain and drug score were reported at two weeks of palliative radiotherapy in 51.5% and 28% of patients, respectively. The patients who finished the assessment had significantly improved overall QoL (P < 0.001).	The patients experienced significant pain response and improved QoL, especially in the first two weeks after radiation. Palliative radiotherapy is a treatment option for patients with painful metastases.	101
Retrospective	EBRT vs Medical or supportive therapy	Prostate, breast, and lung cancer patients with asymptomatic bone metastases	28 vs 143	The median time from the diagnosis of asymptomatic bone metastases to either moderate-to-severe pain or an SRE: 25 months for the untreated patients vs 81 months for the patients receiving EBRT (P < 0.001).	EBRT could be used to delay or prevent late complications of bone metastases that are asymptomatic at the time of diagnosis.	102
Open-label, multicentre, randomised, controlled, phase 2/3 trial	SBRT vs Conventional EBRT	Patients with painful spinal metastases	114 vs 115	Complete response for pain: 40 (35%) of 114 patients in the SBRT vs 16 (14%) of 115 patients in the Conventional EBRT (RR 1.33, 95% CI 1.14-1.55; p = 0.0002).	Spinal SBRT could improve the complete response rate for pain in a specific site of painful spinal metastasis when compared with conventional EBRT.	104
Retrospective	Sr-89	Patients with painful bone metastases in a previously irradiated site	25	24 patients (96.0%) experienced pain relief. One- and 2-year pain progression-free survival rates: 54.5% and 48.4%, respectively.	Sr-89 is an option for patients with a painful bone metastasis in a previously irradiated site.	112
Retrospective	Bisphosphonate therapy ≤24-month group vs >24-month group	Advanced lung cancer patients with bone metastases	187 vs 172	AEs in the ≤ 24-month group were fewer than in the > 24-month treatment group (p = 0.008), and treatment for > 24 months was a potential risk factor for AEs (p = 0.05).	Bisphosphonate treatment beyond 2 years may increase the risk of AEs, but may prolong SRE-free survival early after 24 months, compared with medication administered for ≤ 24 months.	113
Retrospective	Pembrolizumab as first-line therapy vs Second-line therapy or beyond	Advanced NSCLC patients with bone metastases	58 vs 52	Patients (52.7%) received pembrolizumab treatment as first-line therapy vs patients (47.3%) as second-line therapy or beyond: ORR: 41.4% vs 15.4%, P = 0.011; PFS: 9.0 vs 4.0 months, P = 0.004; OS: not reached (NR) vs 11.5 months, P < 0.0001. Bone therapy increased the ORR (34.9% vs 11.1%, P < 0.0001) and prolonged PFS (8.5 vs 2.0 months, P = 0.002).	Pembrolizumab therapy is effective for advanced NSCLC patients with bone metastases, particularly when used in combination with palliative bone radiotherapy or bone-targeted therapy.	129
Retrospective	ICIs	Patients with advanced NSCLC	29	ICIs suppressed the progression of bone metastasis in 21 cases (72.4%). Concomitant therapy with ICIs and denosumab prolonged the OS compared to ICI-only therapy (16.0 months vs. 2.5 months, p < 0.01).	ICIs may successfully suppress the progression of bone metastasis in advanced NSCLC. Systemic treatment of pembrolizumab with denosumab with conservative treatment of bone metastasis could be one of the options in the treatment of advanced NSCLC.	130
Retrospective	ICIs alone vs ICIs + bone-targeted therapy (BTT) vs Other treatments	NSCLC patients with bone metastases	16 vs 30 vs 60	mOS: 15.8 months [95% CI, 8.2-not evaluable (NE)] for the ICI-alone group vs. 21.8 months (95% CI, 14.5-not evaluable) for the ICI + BTT group vs 7.5 (95% CI, 6.1-10.9) months for the group receiving other treatments (p < 0.001).	BTT may have a synergistic effect when used in combination with ICIs, improving patient survival.	131
Retrospective	Denosumab + ICIs (DI) vs Phosphates + ICIs (PI) vs Denosumab + Non-ICIs (DnI) vs Phosphates + Non-ICIs (PnI)	Advanced or recurrent NSCLC patients with bone metastases	40 vs 74 vs 15 vs 42	DI group vs PI group vs DnI group vs PnI group: ORRs: 47.5%, 43.2%, 33.3%, and 40.5%, respectively, p = 0.799; and mPFS: 378, 190, 170, and 172 days, respectively, p = 0.115; SREs: 5%, 10.8%, 13.3%, and 11.9%, respectively, p = 0.733; AEs: 27.5%, 39.2%, 26.7%, and 28.6%, respectively, p = 0.742.	Denosumab exhibits synergistic antitumor efficacy without increasing toxicity when used in combination with ICIs in advanced NSCLC patients with bone metastases.	132
Retrospective	ICIs vs ICIs + Anti-angiogenic agents	Lung cancer patients with bone metastases	53 vs 42	The evaluation of bone metastasis efficacy: higher disease control rate (90.5% vs. 68.6%, p = 0.009), ORR (35.7% vs 24.5%, p = 0.235), and longer median bone PFS (14.3 months vs 8.3 months, p = 0.011) in ICIs + Anti-angiogenic agents group. Lower incidence of SREs in ICIs + anti-angiogenic agents group (28.6% vs 35.8%, p = 0.425).	The combination of ICIs with anti-angiogenic agents may significantly prolong the PFS of bone metastases lesions and potentially decreases SRE.	134

## Abbreviations

NSCLC: non-small cell lung cancer
SREs: skeleton-related events
OS: overall survival
QoL: quality of life
BTAs: bone-targeting agents
BPs: bisphosphonates
EMT: epithelial-mesenchymal transition
LUAD: lung adenocarcinoma
TGF: transforming growth factor
PRAME: preferentially expressed antigen of melanoma
FAIM2: Fas apoptotic inhibitory molecule 2
HIF-1α: hypoxia-inducible factor-1α
TME: tumor microenvironment
BMSCs: bone marrow-derived mesenchymal stem cells
MMPs: matrix metalloproteinases
ECM: extracellular matrix
BSP: bone sialoprotein
CTCs: circulating tumor cells
PD-L1: programmed cell death 1 ligand 1
CXCL12: C-X-C motif chemokine ligand 12
CXCR: C-X-C receptor
sVCAM1: soluble vascular cell adhesion molecule-1
ICAM-1: intercellular adhesion molecule-1
CX3CL1: C-X3-C motif chemokine ligand 1
VBEMCs: vertebral bone marrow endothelial cells
LFA-1: lymphocyte function-associated antigen-1
DTCs: disseminated tumor cells
HSCs: hematopoietic stem cells
NK: natural killer
BRCs: bone remodeling compartments
LIFR: leukaemia inhibitory factor receptor
PTHrP: parathyroid hormone-related protein
HDAC: histone deacetylase
MnO_2_: manganese dioxide
MDSCs: myeloid-derived suppressor cells
Tregs: T- regulatory cells
ICIs: immune checkpoint inhibitors
CTLA-4: cytotoxic T-lymphocyte-associated protein 4
PD-1: programmed cell death 1
CTLs: cytotoxic lymphocytes
RANKL: receptor activator of nuclear factor κB ligand
TNF-α: tumor necrosis factor-α
IFN-γ: interferon-γ
IL: interleukin
PGE2: prostaglandin E2
AdCAR: adapter chimeric antigen receptor
PFS: progression-free survival
ZA: zoledronic acid
IDO1: indoleamine 2, 3-dioxygenase 1
IGF: insulin-like growth factor 1
FGFs: fibroblast growth factors
PDGF: platelet-derived growth factor
BMP2: bone morphogenetic protein 2
M-CSF: macrophage colony-stimulating factor
RANK: receptor activator of nuclear factor κB
OPG: osteoprotegerin
OPN: osteopontin
EGFL6: epidermal growth factor-like domain multiple 6
BMMs: bone marrow mononuclear macrophages
AREG: amphiregulin
ctDNA: circulating tumor DNA
IHC: immunohistochemistry
CaSR: calcium-sensing receptor
TNFSF14: tumor necrosis factor superfamily member 14
EBRT: external beam radiotherapy
SBRT: stereotactic body radiotherapy
^89^Sr: strontium-89
AEs: adverse events
FDA: Food and Drug Administration
ONJ: osteonecrosis of the jaw
BRONJ: bisphosphonate-related osteonecrosis of jaw
ADSCs: adipose-derived stem cells
NMPA: National Medical Products Administration
ORR: objective response rate
DFS: disease-free survival
mOS: median overall survival
